# Mathematical modelling of cell migration: stiffness dependent jump rates result in durotaxis

**DOI:** 10.1007/s00285-019-01344-5

**Published:** 2019-04-10

**Authors:** Adam A. Malik, Philip Gerlee

**Affiliations:** 1 Mathematical Sciences, Chalmers University of Technology, 41296 Göteborg, Sweden; 2 Mathematical Sciences, University of Gothenburg, 41296 Göteborg, Sweden

**Keywords:** Cell migration, Durotaxis, Stochastic model, Jump process, Advection–diffusion equation, 92C17

## Abstract

Durotaxis, the phenomena where cells migrate up a gradient in substrate stiffness, remains poorly understood. It has been proposed that durotaxis results from the reinforcement of focal adhesions on stiff substrates. In this paper we formulate a mathematical model of single cell migration on elastic substrates with spatially varying stiffness. We develop a stochastic model where the cell moves by updating the position of its adhesion sites at random times, and the rate of updates is determined by the local stiffness of the substrate. We investigate two physiologically motivated mechanisms of stiffness sensing. From the stochastic model of single cell migration we derive a population level description in the form of a partial differential equation for the time evolution of the density of cells. The equation is an advection–diffusion equation, where the advective velocity is proportional to the stiffness gradient. The model shows quantitative agreement with experimental results in which cells tend to cluster when seeded on a matrix with periodically varying stiffness.

## Introduction

Cell migration is a process of fundamental importance in a vast range of phenomena. Examples include embryogenesis (Kurosaka and Kashina 2008), during which cells migrate to particular regions to form the structure of an organism, or as part of an immune response (Parkin and Cohen 2001), when macrophages and neutrophils crawl to infected sites, or as part of the wound healing process (Alberts et al. 2002). Another example where cell migration is of great importance is in the growth and spread of cancer (Wang et al. 2005; Yamaguchi et al. 2005), both in local invasion of the surrounding tissue and in formation of metastases.

Two common mechanisms for cell locomotion are “swimming” and “crawling”. Flaggelated cells such as certain bacteria move by rotating a flagella which act as a propeller to drive them forward. A typical example is that of *E*. *coli*, and its motion has been described as a “run and tumble” motion, where it alternates between two phases (Berg et al. 1972). During the running phase it moves in a more or less straight path, followed by a tumbling phase, where it re-orients itself with negligible change in position. The second type of locomotion, crawling, is the process we will focus on in this study. It is a complex phenomena which is often described as a cyclic process, consisting of four distinct phases (Kurosaka and Kashina 2008). The first phase is the polarization phase during which the cell defines its front end. The second is the protrusion phase, in which the cytoskeleton changes shape by extending a protrusion at the leading edge. The third phase is the attachment phase during which it adheres to the substrate on which it is crawling. The last phase is the retraction phase, where the cell pulls itself forward (Alberts et al. 2002; Kurosaka and Kashina 2008), and the trailing edge retracts.

Cell migration that occurs in the human body depends heavily on the properties of the microenvironment, in particular its mechanical properties, and has been the subject of extensive research. The extracellular matrix (ECM) making up the microenvironment is a complex fiber network which is made up of proteoglycans and fibrous proteins, mainly collagens, elastins, fibronectins and liminins (Frantz et al. 2010). It functions as a scaffolding for cells, and plays an important role in proliferation, differentiation and survival of cells (Keogh et al. 2010; Murphy et al. 2012). Abnormalities in the ECM can cause a range of syndromes such as osteogenesis imperfecta and Marfan’s syndrome (Järveläinen et al. 2009). Important properties of the ECM related to cell migration include fiber density (Kaufman et al. 2005; Sander 2014), fiber orientation which can give rise to contact guidance (Schwarz and Bischofs 2005) and cell-substrate adhesiveness which can induce haptotaxis (Carter 1967). In this article we are interested in the impact of ECM elasticity on cell migration, in particular the phenomena known as durotaxis, where cells tend to move towards regions of higher ECM stiffness.

### Durotaxis

Durotaxis was first observed in 2000 by Lo et al. (2000), and has since been observed repeatedly, for different cell types such as fibroblasts (Kuboki et al. 2014), vascular smooth muscle cells (Isenberg et al. 2009), endothelial cells, malignant mammary adenocarcinoma cells (Joaquin et al. 2016), on substrates with different mechanical properties. In the experiment performed by Lo et al. fibroblasts were cultured on a flexible polyacrylamide sheet coated with type I collagen. The sheet consisted of two regions of different rigidity, and cells were approaching the boundary from the soft and from the rigid side. The cell density was low enough for cell–cell interactions to be negligible. The experiment showed that when cells approached the boundary from the soft side, they moved across it into the stiffer region, whereas when they came from the stiffer side the protrusion crossing into the softer side stopped and cells did not cross the boundary. When there is a difference in compliance and the cell exerts forces on the substrate, mass is being pulled toward the stiffer side. However, the authors argue that the skewed mass distribution is not sufficient to explain the phenomena, because the displacements are too small. Instead they proposed that the mechanism is due to cells sensing small changes in stress and strain in the substrate, which is translated into increased traction forces causing a bias in movement direction.

#### Previous models of cell migration

Mathematical modelling has been used to study a range of different interactions between cells and their microenvironment. Some models focus on the subcellular processes such as dynamics of protrusions and stress fibers, and formation of focal adhesions (Harland et al. 2011; Kim et al. 2012, 2015). At a larger spatial scale, the entities of interest are often individual cells and individual fibers, such as in the model of Schlüter et al. (2012). They developed an individual-based model where cells exert forces on the ECM, and assumed that cells had the capacity to realign matrix fibers, modelled as thin cylinders. Their results showed that the cells in their model had a slight preference for stiffer regions. They also found that the cell speed was lower on very stiff matrices. van Oers et al. (2014) developed a hybrid cellular Potts and finite element computational model. The ECM was modelled as a linearly elastic and isotropic medium, and cells were assumed to exert forces on the ECM so it deformed. They also assumed that a strained ECM is stiffer along the orientation of strain than perpendicular to it, a phenomenon known as strain stiffening. Their model also captured durotaxis through an additional term in the energy function, which accounted for the increased probability of cell extension along local strain orientation, and reduced the probability of retraction in that direction.

A mathematical model to investigate durotaxis was developed by Stefanoni et al. (2011). They developed a 2D numerical model based on a modified version of the Langevin equation, where the stochastic force depends on the local stiffness. The cell is assumed to be able to probe the surrounding tissue and sense the local displacement. The stiffest direction is the one where the displacement is smallest. A probability distribution was then constructed for the angular distribution so that the stochastic force is more likely to point in the direction of higher stiffness. However, the stiffness only impacts the angular distribution and not the radial distribution. The authors investigate first the case of a homogeneous and isotropic substrate, where random motility is recovered, and go on to the case of a biphasic domain similar to the one used in Lo et al. and found good agreement with their results.

### The role of adhesion dynamics in durotaxis

During the migration cycle, cells assemble and disassemble focal adhesions. A recent study by Fusco et al. (2017) suggests that the lifetime of adhesion sites depends on the stiffness. The authors investigated the lifetime of focal adhesions on three different substrates with elastic moduli of 30, 200 and 1000 kPa. The average lifetime were shown to be about 6.5, 8 and 12 min respectively.

Joaquin et al. (2016) managed to create a 3D extracellular matrix with varying stiffness, while keeping the other mechanical properties, such as fiber density and protein concetration, constant. They investigate the relationship between substrate stiffness and cell migration velocity, as well as the preference for cells to migrate towards stiffer regions. Their study suggests that cell velocity is uncorrelated with the absolute stiffness, but depends on the stiffness gradient. Their results support a previously suggested theory by Plotnikov and Waterman (2013), which states that there may be a stiffness-dependent mechanism by which focal adhesion sites become reinforced, hence increasing their lifetime, which in turn could cause durotaxis.

A number of computational studies have investigated various aspects of adhesion dynamics on cell migration. For example the model by Ziebert and Aranson (2013), where they accounted for substrate stiffness on the dynamics of adhesion sites. Their model consists of two continuum fields, one for the moving cell boundary and one for the dynamics of the actin cytoskeleton. The dynamics of the number of adhesion contacts is governed by a reaction–diffusion equation and depends on the orientation of the actin cytoskeleton, substrate deformation, and accounts for excluded volume interactions. One of their findings was that their model produced predictions that cells tend to stay on a rigid region, when planted on a region with a discontinuity in stiffness.

Harland et al. (2011) developed a model for sliding adhesion sites and formation and contraction of stress fibers, to investigate the impact of substrate stiffness on durotaxis. Their model showed that in the absence of a stiffness gradient, the expected drift of a cell was zero. When using a linearly increasing elastic modulus, their model predicted the cell drift to be proportional to the ratio of stiffness gradient to the square of the absolute stiffness. They also found that for most stiffness gradients, there exist an optimal stiffness for which the drift is maximized. They use two different assumptions in their model. One is that the number of stress-fibers (and hence adhesion sites) is constant, and that once a fiber contracts enough so its length is reduced below a certain threshold, it is removed and replaced by a new fiber with random orientation. The second assumption is that the formation of stress fibers is a stochastic process, where the formation intensity is stiffness dependent.

Dallon et al. (2013b) introduced a 2D mathematical model for single cell migration, where the cell consists of a nucleus and a number of adhesion sites. The adhesion sites connect to the nucleus through elastic springs, and can be attached or detached to the substrate. Times for attachment and detachment are governed by a Poisson process. When the adhesion site attaches it chooses its position at random. The new position is chosen so that the distance from the cell nucleus is uniformly distributed between $10\, \upmu$m and $15\, \upmu$m, and the direction is chosen uniformly distributed between $-\,\pi /2$ and $\pi /2$ with probability 0.6, and between $\pi /2$ and $3\pi /2$ with probability 0.4. They find that the cell speed is mainly influenced by the mean attachment time, and less by the mean detach time or the strength of the forces the cell exerts on the substrate.

In this work we aim to investigate the impact of stiffness-dependent adhesion lifetimes on the motility of cells, in particular on durotaxis. We do not model subcellular mechanisms, instead we use a model similar to the one introduced by Dallon et al. (2013b) to focus on the individual cell level, where a cell is assumed to consist of a nucleus and fixed number of sites which adhere to the substrate. The adhesion sites connect to the cell nucleus through elastic springs, and the cell moves by updating the position of adhesion sites at random times. To investigate the importance of adhesion lifetimes on durotaxis we assume that the rate at which an adhesion site updates its position depends on the local stiffness of the substrate.

The paper is organized as follows. In Sect. 2 we introduce the mathematical framework for modelling cell migration, in the form of a single-cell stochastic model. In Sect. 3 we analyze the model and derive a macroscopic continuous description for the time evolution of the cell density, and derive an advection–diffusion equation using a diffusion scaling. We then compare the stochastic and deterministic models. To investigate the impact of adhesion lifetimes on directed cell migration, we use biologically relevant parameters and compare our model predictions with experiments in Sect. 4. We summarize our results and discuss their relevance in Sect. 5, as well as possible extensions to our model.

## Model formulation

Our mathematical model of a cell is a 1D model inspired by the model introduced by Dallon et al. (2013b). In the original model a cell was assumed to consist of a nucleus with position *X* and a number of adhesion sites at positions $x_i$ which could be either attached or detached to the substrate. A function $\varPhi _i(t)$ was used to indicate if adhesion site *i* is attached or not, taking value 1 if the site is attached, and 0 if it is detached. The position of a cell *X*, which is the position of the nucleus, satisfied the following equation derived from Newton’s second law of motion under the assumption that the acceleration term can be ignored:

1 $$\begin{aligned} \mu \frac{\mathop {}\!\mathrm {d}X}{\mathop {}\!\mathrm {d}t} = -\sum _{i = 1}^{n} \alpha _i\left( ||X - x_i|| - l_i\right) \frac{X-x_i}{||X-x_i||}\varPhi _i(t), \end{aligned}$$

where $\mu$ is a drag coefficient, $\alpha _i$ the spring coefficient of site *i*, located at position $x_i$. The model also allows for varying rest lengths of the springs, $l_i$. In a later work by Dallon et al. (2013a), they analyzed a simplified version of this model, by informally considering the limit as the spring coefficients become very large. In that case the nucleus reaches its equilibrium position instantaneously, which can be obtained by setting $\mathop {}\!\mathrm {d}X / \mathop {}\!\mathrm {d}t = 0$. In our model we make a number of additional assumptions. We consider a cell in 1D with *n* adhesion sites, located at $x_1, x_2, \ldots , x_n \in \mathbb {R}$. We assume that adhesion sites have detach time 0, meaning that each time an adhesion site detaches, it instantaneously attaches at a new randomly chosen position. At each site we assume that the rest length $l_i$ is zero, and that all spring coefficients are equal. These assumptions together give the position of the cell nucleus as the center of mass of the adhesion sites:

2 $$\begin{aligned} X = \frac{1}{n}\sum _{i = 1}^{n} x_i. \end{aligned}$$

**Fig. 1:**
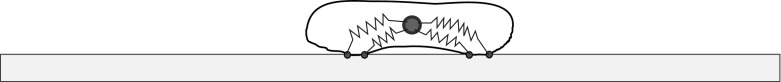
A cartoon figure of our mathematical representation of a cell migrating in 1D space

Each adhesion site updates its position independently, where the waiting times between updates are exponentially distributed. The new position of an adhesion site is generated from a normal distribution centered around the current position of the nucleus, with variance $\sigma ^2$. When an adhesion site updates its position, the nucleus is assumed to change its position instantaneously. A cartoon figure of our mathematical representation of a cell is shown in Fig. 1.

To capture the notion that adhesion lifetimes are dependent on the stiffness of the substrate we assume that cells move on an elastic substrate with elastic modulus given by the function $E = E(x) > 0$, which is assumed to be at least twice continuously differentiable. The elastic modulus determines the mean waiting time until the next update of an adhesion site. For example, if site *i* is located at $x_i$, the time until the next update is exponentially distributed with rate parameter $\lambda _i = G(E(x_i))$, where the functional form of *G* describes how the cells respond to the local stiffness $E(x_i)$, which will be discussed in Sect. 2.1. This means that the average lifetime of site *i*, located at $x_i$, is $1/\lambda _i$. We do not consider the case where the substrate becomes deformed under the force exerted by cells, but plan to investigate this in future work.

A stochastic simulation based on the Gillespie algorithm for the migration of a single cell is given in Algorithm 1.
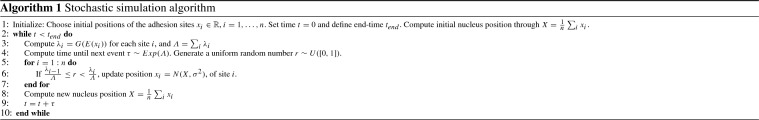


### Stiffness sensing

We investigate two different phenomenologically derived principles for how the stiffness impacts the lifetime of adhesion sites. Both are based on the hypothesis that cells probe the stiffness of the substrate at each adhesion site. If the cell exerts a force at each adhesion site, the site will experience a large displacement in regions where the substrate is soft, and smaller displacements in more rigid regions. We assume that the parameter governing the exponential waiting times is given by some basic rate $\beta \ge 0$, and a contribution which is proportional to the relative difference of the magnitudes of displacement at site *i*, $u_i$, and the average magnitude of displacements at all other sites $\tilde{u} = \frac{1}{n-1}\sum _{j \ne i} |u_j|$, therefore we choose

3 $$\begin{aligned} \lambda _i = \beta + \gamma \frac{|u_i| - |\tilde{u}|}{\sum _j |u_j|}. \end{aligned}$$

The nonnegative parameter $\gamma \ge 0$ influences how much weight is given to the displacement difference. If the displacement $|u_i|$ is large compared to the average at the other sites, $\tilde{u}$, then the site updates more frequently. To compute $u_i$ we assume that the mechanical behaviour of the substrate is that of a Hookean spring of rest length 0 and spring constant given by *E*, so that if the cell exerts a force of magnitude *F*, the magnitude of the displacement is given by

$$\begin{aligned} |u_i| = \left| \frac{F}{E(x_i)}\right| . \end{aligned}$$

In the case of constant stiffness *E*, we have $\lambda _i = \beta$ for all *i*. Moreover, we assume that the force exerted is the same at every adhesion site so the force term *F* can be factored out and incorporated in the parameter $\gamma$. Therefore we simply write $|u_i| = 1/E(x_i)$.

The second principle of stiffness sensing we consider is similar but uses absolute displacement instead of relative displacement, which takes the form

4 $$\begin{aligned} \lambda _i = \beta + \gamma \left( |u_i| - |\tilde{u}|\right) . \end{aligned}$$

We refer to Eq. (3) as using the relative relationship, and (4) as the absolute relationship. We will always choose parameters so that it is ensured that $\lambda _i \ge 0$.

In the next section we derive a population level description in the form of a partial differential equation (PDE), describing the time evolution of the density of non-interacting cells.

## Model analysis

We consider the density of cells *q*(*X*, *t*) at spatial position $X \in \mathbb {R}$ at time $t \ge 0$. By location of a cell we mean the location of its nucleus. Let the function $f_{c}(r\, | \, X)$ be the conditional probability density function (pdf) for a cell making a jump of size *r*, when it is currently residing at position *X*, and assume that the waiting times between adhesion site jumps are exponentially distributed. The density of cells at time $t+ \varDelta t$ is then given by

5 $$\begin{aligned}&q(X,t+\varDelta t) = e^{-\varLambda \varDelta t} q(X,t) + \varLambda \varDelta t e^{-\varLambda \varDelta t}\nonumber \\&\quad \int _{\mathbb {R}}{q(X-r,t) f_c(r\, | \, X-r) \mathop {}\!\mathrm {d}r} + \mathcal {O}(\varDelta t^2), \end{aligned}$$

where $\varLambda$ is the total rate parameter at which the cell nucleus changes position, and is the sum of the individual rate parameters $\varLambda = \lambda _1 + \cdots + \lambda _n$. For both choices of $\lambda _i$ given by (3) and (4), the total rate is $\varLambda = n\beta$ independent of spatial position. The first term on the right hand side describe cells located at *X* at time *t* that did not jump in the interval $[t,t+ \varDelta t]$, and the integral-term correspond to cells being located at $X-r$ at time *t*, and performing a jump of size *r*. The term $\mathcal {O}(\varDelta t^2)$ describe the gain and loss of density whenever a cell exhibit 2 or more jumps in the time interval of size $\varDelta t$. By expanding $e^{-\varLambda \varDelta t}$ in its Taylor series we obtain

$$\begin{aligned} q(X,t+\varDelta t)= & {} (1 - \varLambda \varDelta t + \mathcal {O}(\varDelta t^2))q(X,t) +\, \varLambda \varDelta t (1 - \varLambda \varDelta t + \mathcal {O}(\varDelta t^2)) \\&\quad \int _{\mathbb {R}}{q(X-r,t) f_c(r\, | \, X-r) \mathop {}\!\mathrm {d}r} + \mathcal {O}(\varDelta t^2), \end{aligned}$$

and rearranging gives

$$\begin{aligned} \frac{q(X,t+\varDelta t) - q(X,t)}{\varDelta t}= & {} q(X,t)(-\varLambda + \mathcal {O}(\varDelta t)) +\, \varLambda (1 - \mathcal {O}(\varDelta t))\\&\quad \int _{\mathbb {R}}q(X-r,t)f_c(r \, | \, X-r) \mathop {}\!\mathrm {d}r + \mathcal {O}(\varDelta t^2). \end{aligned}$$

We now take the limit $\varDelta t \rightarrow 0$ and obtain the partial integro-differential equation, often called a transport equation:

6 $$\begin{aligned} \frac{\partial q}{\partial t}(X,t) = -\varLambda q(X,t) + \varLambda \int _{\mathbb {R}}{q(X-r,t) f_c(r\, | \, X-r) \mathop {}\!\mathrm {d}r}. \end{aligned}$$

Notice that the rate parameter $\varLambda$ is the sum of all individual rate parameters $\lambda _i$, as it describes the rate at which any event occurs. In what follows the rate parameters $\lambda _i$ will be spatially varying.

### The distribution of nucleus jumps

We now simplify our analysis by considering only two adhesion sites, located at $x_1$ and $x_2$. Since the nucleus is located at the center of mass of $x_1$ and $x_2$, that is $X = (x_1 + x_2)/2$, we can express the position of the two adhesion sites as

$$\begin{aligned} x_1 = X - Y, \,\,\, x_2 = X + Y, \end{aligned}$$

where 2*Y* is the distance between the two adhesion sites, and $Y \ge 0$.

It can be shown (see “Appendix C” for details) that in the stochastic model of motion described in Sect. 2, the distance *Y* converges to a random variable distributed half-normal with mean $\sigma \sqrt{2/3\pi }$ and variance $\sigma ^2\left( 1-2/\pi \right) /3$, whenever the new position of an adhesion site is normally distributed around the current nucleus position with variance $\sigma ^2$. We denote the pdf of *Y* with $f_Y$, given by

$$\begin{aligned} f_{Y}(y) = \frac{\sqrt{6}}{\sigma \sqrt{\pi }} e^{-\frac{y^2}{2\sigma ^2/3}}, y\ge 0, \end{aligned}$$

and proceed to compute the pdf governing nucleus jumps through

7 $$\begin{aligned} f_{C}(r\, | \, X) = \int _{0}^{\infty }{f_{C \, | \, Y}(r \, | \, X, y) f_{Y}(y)\mathop {}\!\mathrm {d}y}, \end{aligned}$$

where $f_{C}(r\, | \, X, y)$ is the conditional probability density function of a nucleus jump, conditioned on the current nucleus position *X*, and the current distance *y*.

Assume that the current nucleus position *X* is known, as well as the current distance *y*. The nucleus then jumps as a result of either site $i = 1$ performing a jump, or the site $i = 2$ performing a jump. Whenever the left site makes a jump, the nucleus is on average displaced to the right, and whenever the right site jumps, the nucleus is on average displaced to the left. In both cases the average displacement of the nucleus is exactly half as much as the jump of an individual adhesion site. Therefore, the probability density $f_C(r \, | \, X, y)$ is a mixture-distribution of two components, given by

8 $$\begin{aligned} f_{C \, | \, Y}(r \, | \,X, y) = w_1(X-y) \frac{2}{\sqrt{\pi \sigma ^2}}e^{-\frac{2\left( r-y/2\right) ^2}{\sigma ^2}} + w_2(X+y)\frac{2}{\sqrt{\pi \sigma ^2}}e^{-\frac{2\left( r + y/2\right) ^2}{\sigma ^2}}. \end{aligned}$$

In other words, it is a sum of the pdfs of two normal distributions with means *y* / 2 and $-y/2$ respectively, and variance $\sigma ^2/4$. The first component in the mixture comes from the case that site $i = 1$ updates, and the second component from the case that site $i = 2$ updates. The weights of the mixture $w_1$ and $w_2$ are given by

9 $$\begin{aligned} w_1(X-y) = \frac{\lambda _1}{\lambda _1 + \lambda _2}, \,\,\, w_2(X+y) = \frac{\lambda _2}{\lambda _1 + \lambda _2}. \end{aligned}$$

It is important to observe that the weights in the mixture are functions of the positions $x_1$ and $x_2$, or equivalently as formulated above, functions of the current nucleus position *X* and the distance *y* between the sites.

To summarize, the probability density function governing the size of a nucleus jump $f_C(r\,|\, X)$, when the nucleus is located at *X*, is given as a mixture of two components, where each component comes from whether the first or second adhesion site updates. The weights depend on where the sites are located.

To proceed we use $\lambda _i$ defined in (3) and (4) described in Sect. 2.1, and we derive an advection–diffusion equation for the cell density using a large time diffusion scaling.

### Large time diffusion approximation

We now consider the case where the rate of updates is given by Eq. (3), namely

$$\begin{aligned} \lambda _i = \beta + \gamma \left( \frac{|u_i|-|\tilde{u}|}{\sum _{j =1}^n |u_j|} \right) . \end{aligned}$$

For two sites $x_1 = X - y$ and $x_2 = X + y$ this gives

$$\begin{aligned} \lambda _1&= \beta + \gamma \left( \frac{|u_1|-|u_2|}{|u_1| + |u_2|} \right) , \\ \lambda _2&= \beta - \gamma \left( \frac{|u_1|-|u_2|}{|u_1| + |u_2|} \right) , \end{aligned}$$

and the total rate is then given by

$$\begin{aligned} \varLambda (X) = \lambda _1 + \lambda _2 = 2\beta . \end{aligned}$$

We now substitute $u_i = 1/E(x_i)$ and simplify to get

$$\begin{aligned} \lambda _1&= \beta + \gamma \left( \frac{E(x_2)-E(x_1)}{E(x_1) + E(x_2)} \right) = \beta + \gamma \left( \frac{E(X+y)-E(X-y)}{E(X-y) + E(X+y)} \right) , \\ \lambda _2&= \beta - \gamma \left( \frac{E(x_2)-E(x_1)}{E(x_1) + E(x_2)} \right) =\beta - \gamma \left( \frac{E(X+y)-E(X-y)}{E(X-y) + E(X+y)} \right) . \end{aligned}$$

We now expand *E* in its Taylor series around *X* to obtain

$$\begin{aligned} \lambda _1&= \beta + \gamma \frac{yE'(X)}{E(X)} + \gamma \sum _{i = 3,5,7,\ldots } T_i(X)y^{i},\\ \lambda _2&= \beta - \gamma \frac{yE'(X)}{E(X)} - \gamma \sum _{i = 3,5,7,\ldots } T_i(X)y^{i}, \end{aligned}$$

where the term $T_i$ is the *i*th term in the Taylor series of *E*, depending only on *X*, and the sum containing only odd indices because all even terms cancel in the series. Using (8) and (9) in Eq. (7), we thus obtain the conditional jump distribution as

$$\begin{aligned} f_{C}(r,X)&= \int _{0}^{\infty }\left( w_1(X-y)\frac{1}{\sqrt{2\pi \sigma ^2/4}}e^{-\frac{(r-y/2)^2}{2\sigma ^2/4}}\right) f_{Y}(y) \mathop {}\!\mathrm {d}y \\&\quad + \int _{0}^{\infty }\left( w_2(X+y)\frac{1}{\sqrt{2\pi \sigma ^2/4}}e^{-\frac{(r+y/2)^2}{2\sigma ^2/4}} \right) f_{Y}(y) \mathop {}\!\mathrm {d}y \\&=\int _{0}^{\infty } \frac{1}{2\beta }\left( \left( \beta + \gamma \frac{yE'(X)}{E(X)}\right) \frac{1}{\sqrt{2\pi \sigma ^2/4}}e^{-\frac{(r-y/2)^2}{2\sigma ^2/4}}\right) f_{Y}(y) \mathop {}\!\mathrm {d}y \\&\quad +\int _{0}^{\infty } \frac{1}{2\beta }\left( \left( \beta - \gamma \frac{yE'(X)}{E(X)}\right) \frac{1}{\sqrt{2\pi \sigma ^2/4}}e^{-\frac{(r+y/2)^2}{2\sigma ^2/4}} \right) f_{Y}(y) \mathop {}\!\mathrm {d}y \\&\quad + \frac{1}{2\beta }\sum _{i = 3,5,\ldots }\int _{0}^{\infty }T_i(X)y^i \left( \frac{1}{\sqrt{2\pi \sigma ^2/4}}e^{-\frac{(r-y/2)^2}{2\sigma ^2/4}}\right) f_{Y}(y) \mathop {}\!\mathrm {d}y \\&\quad - \frac{1}{2\beta }\sum _{i = 3,5,\ldots }\int _{0}^{\infty }T_i(X)y^i \left( \frac{1}{\sqrt{2\pi \sigma ^2/4}}e^{-\frac{(r+y/2)^2}{2\sigma ^2/4}} \right) f_{Y}(y) \mathop {}\!\mathrm {d}y \\&= \frac{1}{2\beta }\left( 2\beta +\gamma \frac{rE'(X) }{E(X)} \right) \frac{1}{\sqrt{2\pi \sigma ^2/3}} e^{-\frac{r^2}{2\sigma ^2/3}}\\&\quad + \frac{1}{2\beta } \sum _{i = 3,5,\ldots } \int _{0}^{\infty } T_i(X)y^i\left( \frac{1}{\sqrt{2\pi \sigma ^2/4}}e^{-\frac{(r-y/2)^2}{2\sigma ^2/4}}\right) \frac{\sqrt{6}}{\sigma \sqrt{\pi }}e^{-\frac{y^2}{2\sigma ^2/3}} \mathop {}\!\mathrm {d}y \\&\quad - \frac{1}{2\beta } \sum _{i = 3,5,\ldots } \int _{0}^{\infty } T_i(X)y^i\left( \frac{1}{\sqrt{2\pi \sigma ^2/4}}e^{-\frac{(r+y/2)^2}{2\sigma ^2/4}} \right) \frac{\sqrt{6}}{\sigma \sqrt{\pi }}e^{-\frac{y^2}{2\sigma ^2/3}} \mathop {}\!\mathrm {d}y. \end{aligned}$$

One can see that the last two terms of sums of integrals can be written as:

$$\begin{aligned} \frac{1}{2\beta }\frac{1}{\sqrt{2\pi \sigma ^2}}e^{-\frac{r^2}{\sigma ^2/3}} \frac{\sqrt{6}}{\sigma \sqrt{\pi }}\sum _{i = 3,5,\ldots }T_i(X) \int _{-\infty }^{\infty }e^{-\frac{(y-r/2)^2}{2\sigma ^2/4}} y^i \mathop {}\!\mathrm {d}y. \end{aligned}$$

That is, we obtain the sum of higher order non-central moments of the normal distribution with mean *r* / 2 and variance $\sigma ^2/4$. We know that the *i*th moment of a normal distribution with mean *r* / 2 and variance $\sigma ^2/4$ is a sum of the form

$$\begin{aligned} \frac{1}{\sqrt{2\pi \sigma ^2/4}}\int _{-\infty }^{\infty }e^{-\frac{(y-r/2)^2}{2\sigma ^2/4}} y^i \mathop {}\!\mathrm {d}y = \sum _{j = 0}^{\lfloor i/2 \rfloor } c_j \sigma ^{2j}\left( \frac{r}{2}\right) ^{i-2j}, \end{aligned}$$

where the coefficient $c_j$ is given by the product of a binomial coefficient and the double factorial:

$$\begin{aligned} c_j = \left( {\begin{array}{c}i\\ 2j\end{array}}\right) (2j-1)!! \end{aligned}$$

We therefore obtain the final result of the form

$$\begin{aligned}&= \frac{1}{\sqrt{2\pi \sigma ^2}}e^{-\frac{r^2}{\sigma ^2/3}} \sqrt{3}\sum _{i = 3,5,\ldots }T_i(X) \frac{1}{\sqrt{2\pi \sigma ^2/4}}\int _{-\infty }^{\infty }e^{-\frac{(y-r/2)^2}{2\sigma ^2/4}} y^i \mathop {}\!\mathrm {d}y \\&= \frac{1}{\sqrt{2\pi \sigma ^2}}e^{-\frac{r^2}{\sigma ^2/3}} \sum _{i=3,5,\ldots }T_i(X) \sum _{j = 0}^{\lfloor i/2\rfloor } c_j \sigma ^{2j}\left( \frac{r}{2}\right) ^{i-2j}, \end{aligned}$$

where we have incorporated the constant $\sqrt{3}$ into the constant $c_j$. To summarize, the jump distribution weighted by the total jump rate $\varLambda (X) = \varLambda = 2\beta$, as formulated in the transport equation (6), is given by

10 $$\begin{aligned} \varLambda f_C(r,X)&= \frac{1}{\sqrt{2\pi \sigma ^2/3}} e^{-\frac{r^2}{2\sigma ^2/3}} \left( 2\beta +\gamma \frac{rE'(X) }{E(X)} \right) \end{aligned}$$

11 $$\begin{aligned}&\quad + \frac{1}{\sqrt{2\pi \sigma ^2/3}} e^{-\frac{r^2}{2\sigma ^2/3}} \sum _{i=3,5,\ldots }T_i(X) \sum _{j = 0}^{\lfloor i/2\rfloor } c_j \sigma ^{2j}\left( \frac{r}{2}\right) ^{i-2j}. \end{aligned}$$

We now insert this expression into Eq. (6) and obtain

$$\begin{aligned}&\frac{\partial q}{\partial t}(X,t) = -\varLambda q(X,t) + \varLambda \int _{\mathbb {R}}{q(X-r,t) f_c(r\, | \, X-r) \mathop {}\!\mathrm {d}r} \\&= -\varLambda q(X,t) + \int _{\mathbb {R}}{q(X-r,t) \frac{1}{\sqrt{2\pi \sigma ^2/3}} e^{-\frac{r^2}{2\sigma ^2/3}} \left( 2\beta +\gamma \frac{rE'(X-r) }{E(X-r)} \right) \mathop {}\!\mathrm {d}r} \\&\quad \quad + \int _{\mathbb {R}}{q(X-r,t) \frac{1}{\sqrt{2\pi \sigma ^2/3}} e^{-\frac{r^2}{2\sigma ^2/3}} \sum _{i=3,5,\ldots }T_i(X-r) \sum _{j = 0}^{\lfloor i/2\rfloor } c_j \sigma ^{2j}\left( \frac{r}{2}\right) ^{i-2j} \mathop {}\!\mathrm {d}r}. \end{aligned}$$

We now proceed with the following rescaling for jump-size and time:

12 $$\begin{aligned} \sigma = \varepsilon \hat{\sigma }, t = \frac{1}{\varepsilon ^2}\tau , \end{aligned}$$

for a small parameter $\varepsilon$. This corresponds to a cell which performs small jumps with high frequency. Writing $q(X,t) = q\left( X,\frac{1}{\varepsilon ^2}\tau \right) = \hat{q}\left( X,\tau \right)$ the equation is

$$\begin{aligned}&\varepsilon ^2 \frac{\partial \hat{q}}{\partial \tau }(X,\tau ) = -\varLambda \hat{q}(X,\tau ) \\&\quad + \int _{\mathbb {R}}{\hat{q}(X-r,\tau ) \frac{1}{\varepsilon } \frac{1}{\sqrt{2\pi \hat{\sigma }^2/3}} e^{-\frac{(r/\varepsilon )^2}{2\hat{\sigma }^2/3}} \left( 2\beta +\gamma \frac{rE'(X-r) }{E(X-r)} \right) \mathop {}\!\mathrm {d}r} \\&\quad + \int _{\mathbb {R}}{\hat{q}(X-r,\tau ) \frac{1}{\varepsilon }\frac{1}{\sqrt{2\pi \hat{\sigma }^2/3}} e^{-\frac{(r/\varepsilon )^2}{2\hat{\sigma }^2/3}} \sum _{i=3,5,\ldots }T_i(X-r) \sum _{j = 0}^{\lfloor i/2\rfloor } c_j \varepsilon ^{2j} \hat{\sigma }^{2j}\left( \frac{r}{2}\right) ^{i-2j} \mathop {}\!\mathrm {d}r}, \end{aligned}$$

and we perform the change of variables, $r = s\varepsilon$ to obtain

$$\begin{aligned}&\varepsilon ^2 \frac{\partial \hat{q}}{\partial \tau }(X,\tau ) = -\varLambda \hat{q}(X,\tau ) \\&\quad + \int _{\mathbb {R}}{\hat{q}(X-s\varepsilon ,\tau ) \frac{1}{\sqrt{2\pi \hat{\sigma }^2/3}} e^{-\frac{s^2}{2\hat{\sigma }^2/3}} \left( 2\beta +\gamma s\varepsilon \frac{E'(X-s\varepsilon ) }{E(X-s\varepsilon )} \right) \mathop {}\!\mathrm {d}s} \\&\quad + \int _{\mathbb {R}}{\hat{q}(X-s\varepsilon ,\tau ) \frac{1}{\sqrt{2\pi \hat{\sigma }^2/3}} e^{-\frac{s^2}{2\hat{\sigma }^2/3}} \sum _{i=3,5,\ldots }T_i(X-s\varepsilon ) \sum _{j = 0}^{\lfloor i/2\rfloor } c_j \varepsilon ^{i} \hat{\sigma }^{2j}\left( \frac{s}{2}\right) ^{i-2j} \mathop {}\!\mathrm {d}s}. \end{aligned}$$

We now proceed with Taylor expanding the function $\hat{q}(X-s\varepsilon ,\tau )$ in space around *X*. Also notice that the last term containing the higher order moments of the normal distribution is of at least order $\varepsilon ^3$, and will therefore be neglected in the limit $\varepsilon \rightarrow 0$. For the sake of clarity we keep the second integral, but write it compactly as $\mathcal {O}(\varepsilon ^3)$. Our equation now is

$$\begin{aligned} \varepsilon ^2 \frac{\partial \hat{q}}{\partial \tau }(X,\tau )= & {} -\varLambda \hat{q}(X,\tau ) \\&+ \int _{\mathbb {R}}{\hat{q}(X,\tau ) \frac{1}{\sqrt{2\pi \hat{\sigma }^2/3}} e^{-\frac{s^2}{2\hat{\sigma }^2/3}} \left( 2\beta +\gamma s\varepsilon \frac{E'(X-s\varepsilon ) }{E(X-s\varepsilon )} \right) \mathop {}\!\mathrm {d}s} \\&-\int _{\mathbb {R}}{s\varepsilon \frac{\partial \hat{q}}{\partial X}(X,\tau ) \frac{1}{\sqrt{2\pi \hat{\sigma }^2/3}} e^{-\frac{s^2}{2\hat{\sigma }^2/3}} \left( 2\beta +\gamma s\varepsilon \frac{E'(X-s\varepsilon ) }{E(X-s\varepsilon )} \right) \mathop {}\!\mathrm {d}s} \\&+\int _{\mathbb {R}}{\frac{s^2\varepsilon ^2}{2} \frac{\partial ^2 \hat{q}}{\partial X^2}(X,\tau ) \frac{1}{\sqrt{2\pi \hat{\sigma }^2/3}} e^{-\frac{s^2}{2\hat{\sigma }^2/3}} \left( 2\beta +\gamma s\varepsilon \frac{E'(X-s\varepsilon ) }{E(X-s\varepsilon )} \right) \mathop {}\!\mathrm {d}s} \\&+ \mathcal {O}(\varepsilon ^3). \end{aligned}$$

We now assume that the stiffness *E* is varying slowly in comparison to the typical size of a cell, and expand the fraction $E'/E$ in its Taylor series around *X*:

$$\begin{aligned} \frac{E'(X-s\varepsilon )}{E(X-s\varepsilon )} = \frac{E'(X)}{E(X)} + s\varepsilon \frac{E'(X)^2 - E(X)E''(X)}{E(X)^2} + \mathcal {O}(\varepsilon ^2). \end{aligned}$$

Computing the integrals of our equation, we obtain the final result

$$\begin{aligned} \varepsilon ^2 \frac{\partial \hat{q}}{\partial \tau }&= -\varepsilon ^2 \hat{q}(X,\tau )\left( \frac{E(X)E''(X) - E'(X)^2}{E(X)^2}\right) \gamma \frac{\hat{\sigma }^2}{3} \\&\quad \quad -\,\varepsilon ^2 \frac{\partial \hat{q}}{\partial X}(X,\tau )\left( \frac{E'(X)}{E(X)} \right) \gamma \frac{\hat{\sigma }^2}{3} + \varepsilon ^2 \frac{\partial ^2 \hat{q}}{\partial X^2}\beta \frac{\hat{\sigma }^2}{3} + \mathcal {O}(\varepsilon ^3). \end{aligned}$$

Dividing through by $\varepsilon ^2$ and taking the limit $\varepsilon \rightarrow 0$, we obtain the equation

13 $$\begin{aligned} \frac{\partial \hat{q}}{\partial \tau } = -\gamma \frac{\hat{\sigma }^2}{3}\frac{\partial }{\partial X}\left( \frac{E'(X)}{E(X)} \hat{q}(X,\tau ) \right) + \beta \frac{\hat{\sigma }^2}{3}\frac{\partial ^2 \hat{q}}{\partial X^2}, \end{aligned}$$

after writing the first two terms as a single derivative of a product.

We can perform the exact same analysis when using the absolute relationship (4), with the same assumption on a slowly varying function *E*, and the same diffusion-scaling to obtain the equation

14 $$\begin{aligned} \frac{\partial \hat{q}}{\partial \tau } = -\gamma \frac{\hat{\sigma }^2}{3}\frac{\partial }{\partial X}\left( \frac{2E'(X)}{E^2(X)} \hat{q}(X,\tau ) \right) + \beta \frac{\hat{\sigma }^2}{3}\frac{\partial ^2 \hat{q}}{\partial X^2}, \end{aligned}$$

which is different only in the advective velocity being $2E'(X)/E^2(X)$ instead of $E'(X)/E(X)$. For details regarding the derivation of Eq. (14), see “Appendix A”.

### Comparison between stochastic simulations and PDE model

We now compare the stochastic simulation to the solutions of the PDE models. We compute the trajectories of 1500 cells using the stochastic simulation, compute the occupancy in discretized intervals of the spatial domain, and normalize so that the total cell density is 1. The PDE model is solved using a Crank–Nicolson finite difference scheme (see “Appendix B” for details) using the same spatial discretization, and total density chosen to be 1.

We compare the models for a linearly increasing stiffness function $E(x) = 3 + 0.9x$, on the domain $\varOmega = [-\,3,3]$, with parameters $\sigma = 0.05$, $\beta = 0.5$, $\gamma = 1$ for $t \in [0,400]$. In the stochastic simulations all cells are starting out at the origin, with both adhesion sites located at the origin, and in the PDE model we use a Dirac-type initial condition, located at the origin. In the stochastic simulation, cells are not able to migrate past the domain boundaries at $\pm \, 3$. This is implemented by checking if a cell is attempting to move outside the domain. If it is, it simply does not move. In the PDE model we use zero-flux boundary conditions.

**Fig. 2:**
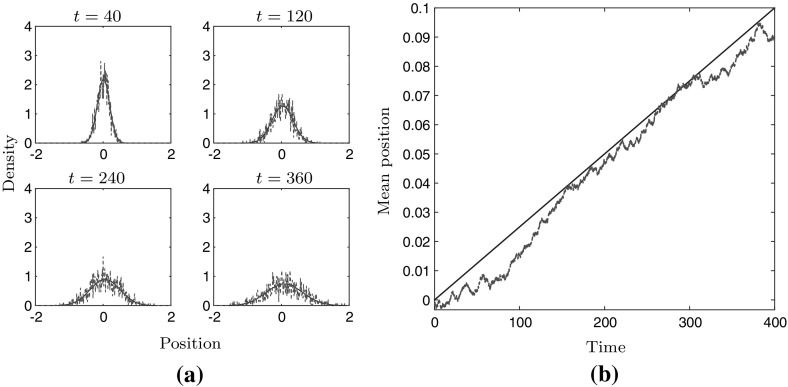
Numerical comparison between stochastic model (red) and PDE model (blue) when using the relative relationship (3). Panel **a** shows the cell density at four instances of time. Panel **b** shows the mean position of the cell obtained from the stochastic model and the PDE model. $\sigma = 0.05$, $\beta = 0.5$, $\gamma = 1$, $E(x) = 3 + 0.9x$ on the domain $[-\,3,3]$ for 400 units of time (colour figure online)

**Fig. 3:**
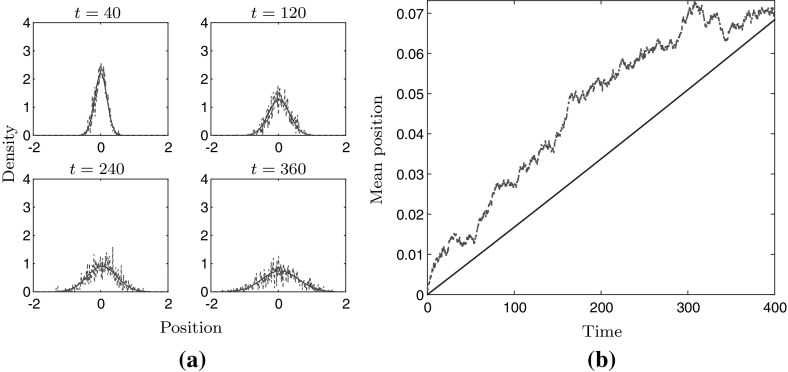
Numerical comparison between stochastic model (red) and PDE model (blue) when using the absolute relationship (4). Panel **a** shows the time evolution at 4 time instances. Panel **b** shows the mean position of the cell obtained from the stochastic and PDE model. $\sigma = 0.05$, $\beta = 0.5$, $\gamma = 1$, $E(x) = 3 + 0.9x$ on the domain $[-\,3,3]$ for 400 units of time (colour figure online)

Figure 2a shows the cell density at four different instances in time, the red dashed line corresponding to the stochastic simulation and the blue line to the solution of PDE (13), when using the relative relationship (3). A comparison between the mean position of a cell is shown in Fig. 2b. Figure 3a, b shows the same comparison when using the absolute relationship (4).

In both cases the stochastic simulation and the solution of the PDE show good agreement.

## Periodically varying stiffness result in cell clusters

In this section we use our model to investigate if cells cluster on stiff regions, when seeded uniformly at random on a substrate with a sinusoidal variation in stiffness. This is motivated by the study performed by Joaquin et al. (2016), where the matrix was modified so that there was a spatial variation in the stiffness, in the form of a sine wave. In the experiments four different cell types were used, and all four cell types formed clusters on the stripes of high stiffness but had different final morphologies. From a correlation analysis they concluded that the stiffness gradient was the driving factor in this durotactic phenomena, and not the absolute stiffness.

**Fig. 4:**
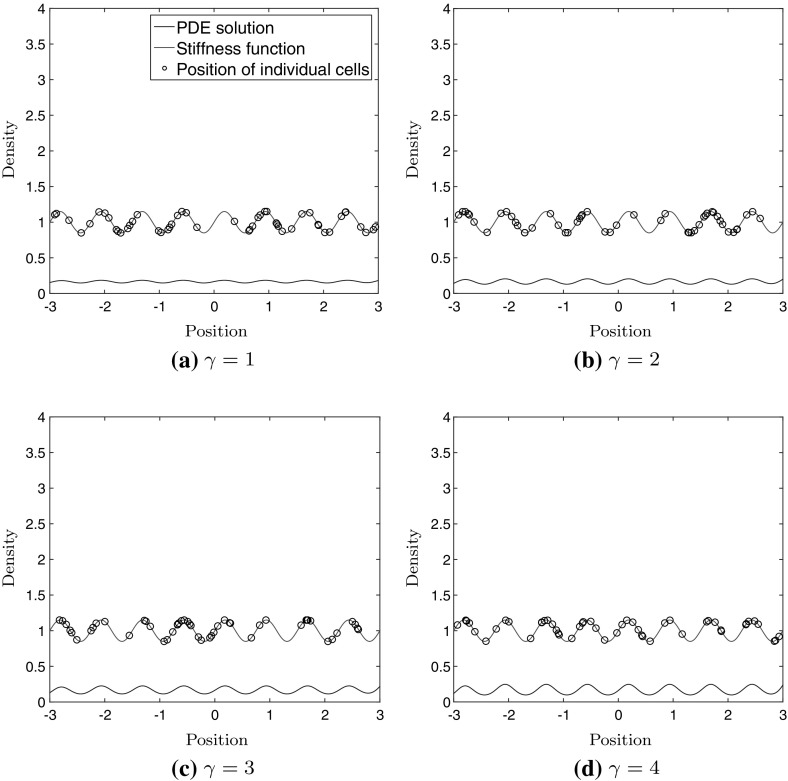
Stochastic simulation of 50 cells, and the solution to the PDE model (13) after 24 h, with uniformly distributed cells at time $t = 0$. The stiffness function is superimposed to illustrate the position of the regions of high and low stiffness

We now want to investigate if our model will produce the same behaviour, namely that cells cluster on regions of high stiffness. To do so we need to find appropriate parameter values. Recall that the mean distance between two sites is a random variable with mean $\sigma \sqrt{2/3\pi }$. Now we wish to choose the cell sizes to be on average $20 \mu$m, which holds if we choose

15 $$\begin{aligned} \sigma \sqrt{\frac{2}{3\pi }} = 20\,\upmu \text{ m }, \end{aligned}$$

which requires us to set $\sigma \approx 40\,\upmu \text{ m } = 0.04\, \text{ mm }$. We choose the time to be measured in hours and the rates to have the unit updates per hour. The stiffness function is chosen to match the stiffness profile from the experiments, and is given by

16 $$\begin{aligned} E(x) = 1 + 0.15\sin \left( \frac{8\pi x}{3}\right) , \end{aligned}$$

and the domain to be $\varOmega = [-\,3, 3] \text{ mm }$, so there are a total of 8 peaks of high stiffness in the domain. We use uniformly distributed initial positions of cells in the stochastic simulation, and constant cell density throughout the domain in the PDE model, normalized so the total density is 1.

For our choice of $\sigma$, the nucleus jump is on average of size $9.21\, \upmu$m, and the rate at which it jumps is $2\beta$, in the absence of a stiffness gradient, so choosing $\beta = 1$ results in cells which move about $20\,\upmu$m per hour on average, which is in a realistic range for many cell types.

Figure 4 show the results of a simulation with 50 cells using the relative relationship (3) along with the solution to the PDE model (13) after 24 h, for four different values of $\gamma \in \{1,2,3,4\}$, with unit $\hbox {h}^{-1}$ in the case of using the relative relationship, and $\text{ mm }^{-1}\hbox {h}^{-1}$ in the case of using the absolute relationship. It can be seen that for larger values of $\gamma$, the cells tend to cluster at the peaks. This is expected since the parameter $\gamma$ governs how big impact the difference in displacement (relative or absolute) has on the update rates. The stiffness function is shown in the plots to illustrate where the peaks are located, and the position of cells are plotted on this superimposed curve to better illustrate where they are located. Note however that the cells move on a 1-dimensional line.

**Fig. 5:**
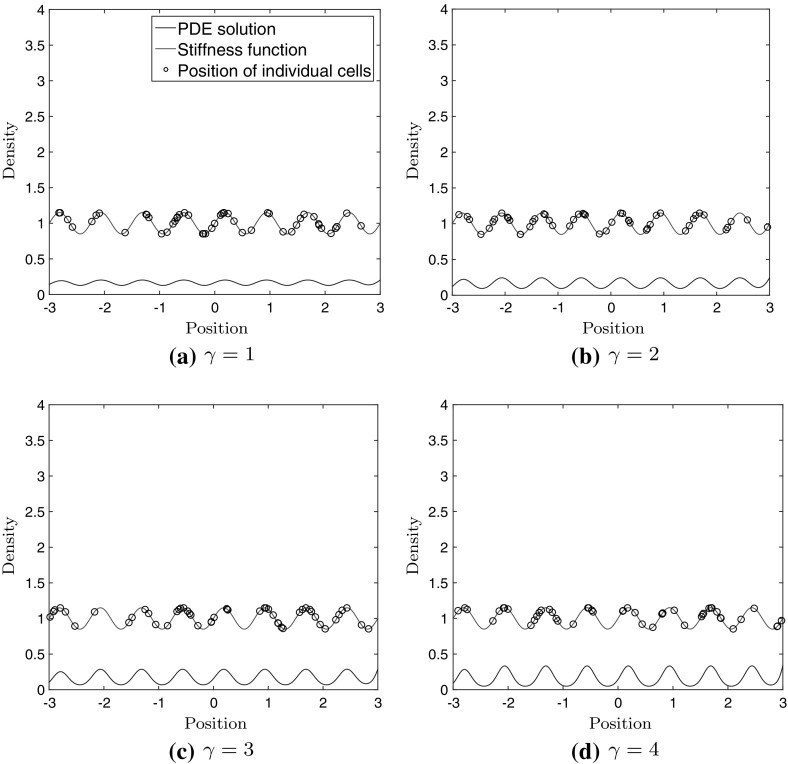
Stochastic simulation of 50 cells, and the solution to the PDE model (14) after 24 h, with uniformly distributed cells at time $t = 0$. The stiffness function is superimposed to illustrate the position of the regions of high and low stiffness

Figure 5 shows the same comparison when using the absolute relationship (4) and the corresponding PDE model (14).

To better understand how the lifetime of adhesion sites impact clustering we also investigate the difference between lifetimes of the two adhesion sites, for $\gamma \in \{0, 1, 2, 3, 4\}$. We do this by computing the minimum and maximum of $\lambda _1$ and $\lambda _2$ for each cell as it is moving around, and average over 2000 simulations. The results can be seen in the table below. This means that for example, in the case of using $\gamma = 4$ the site that updates most frequently does so on average 15% more often than the site that updates less frequently (Table 1).

**Table 1:** Table of the average low and average high update frequencies, averaged over 50 cells for 24 h of migration

$\gamma$	$\lambda _{low}$	$\lambda _{high}$
(*a*) *Relative relationship*		
0	0.5	0.5
1	0.4907	0.5093
2	0.4828	0.5172
3	0.4750	0.5250
4	0.4650	0.5348
(*b*) *Absolute relationship*		
0	0.5	0.5
1	0.4821	0.5179
2	0.4648	0.5352
3	0.4445	0.5555
4	0.4323	0.5677

We now introduce a way to measure to which degree cells cluster by defining the following quantities. We define *H* to be the part of our spatial domain where the stiffness is higher than the average stiffness over the entire domain, and *L* to be the part where the stiffness is lower than the average. Then we define the total cell density on high stiffness and low stiffness regions, at time *t*, through

17 $$\begin{aligned} N_{H}(t) = \int _{H} q(x,t) \mathop {}\!\mathrm {d}x, \qquad N_L(t) = \int _{L} q(x,t) \mathop {}\!\mathrm {d}x, \end{aligned}$$

respectively. We consider the ratio

18 $$\begin{aligned} R(t) = N_H(t)/(N_H(t) + N_L(t)), \end{aligned}$$

i.e. ratio of cell density located on a stiff region, to the total cell density. Notice that this measure ranges from 0 to 1, and takes the value 1/2 in the case that the cells are uniformly spread between the soft and stiff regions.

**Fig. 6:**
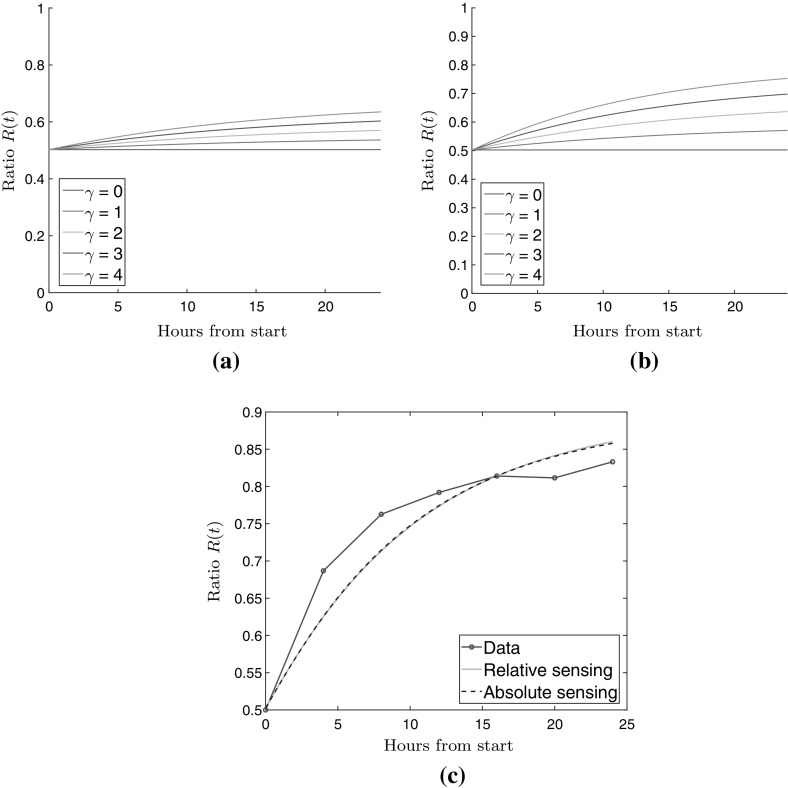
Ratio of total cell density on stiff regions to total cell density on soft regions over 24 h. Ratio computed using (18), using the relative relationship in panel **a** and the absolute relationship in panel **b** for $\gamma = 0,1,2,3,4$. Panel **c** shows comparison between experiments carried out by Joaquin et al. (2016) and the two PDE models when $\gamma$ is fitted using the least-squares method

Figure 6a, b show the evolution of the ratio *R*(*t*) for different values of $\gamma$, corresponding to the experiments shown in Figures 4 and 5. The ratio is computed using the PDE model. In order to compare our predictions with the experiments carried out by Joaquin et al. (2016), we analyzed a supplementary video published with that study. Images were extracted at 4, 8, 12, 16, 20 and 24 h and segmented using Otsu’s method implemented in MATLAB (Otsu 1979). The total cell area was calculated in the high and low stiffness regions. From these quantities we calculated the fraction of the cell population being located on the stiff region for each image, corresponding to (18). The resulting data is shown in Fig. 6c where we have fit the parameter $\gamma$ using least squares in our two PDE models and calculated the ratio defined in (18). It can be seen that the two PDE models provide near identical predictions, but use different values of $\gamma$. When fitting the PDE model based on the relative sensing we obtained $\gamma = 13.28$ and when using the model based on the absolute sensing we obtained $\gamma = 6.5$.

## Conclusion

The mechanisms causing durotaxis remain poorly understood. A hypothesis has been proposed, that through repeated tugging, cells probe the stiffness of their surrounding and adhesion sites on stiff regions become reinforced (Joaquin et al. 2016; Plotnikov and Waterman 2013). In this work we developed a mathematical model for investigating directed cell migration, under the assumption that adhesion sites become reinforced on stiff regions. The model assumes that a cell migrates by updating the position of its adhesion sites at random times, and a new position is chosen randomly around its current nucleus position. The cells are assumed to be able to sense the local stiffness by exerting a force at every adhesion site. We propose two sensing mechanisms. The first is based on the relative displacement at adhesion sites, and the second based on the absolute displacement. To model reinforcement we assume that adhesion sites where the displacement is small update less frequently than sites where the displacement is large.

From the individual based model we derived a continuous description in the form of a partial differential equation for the density of cells. The resulting PDEs are advection diffusion equations, where the cells are predicted to move in the direction of increasing stiffness. In the case of assuming a relative sensing mechanism cells move with a velocity proportional to ratio of stiffness gradient to absolute stiffness, and in the case of assuming an absolute sensing mechanism, the velocity is proportional to the ratio of stiffness gradient to the square of absolute stiffness. The stochastic and deterministic models showed good agreement for the length and time scales typically observed in experiments.

Another type of taxis observed in biology is chemotaxis, where an organism responds to the concentration of some chemical to which it is chemosensitive. A continuum description is given by the classical chemotaxis equation (Erban and Othmer 2004) for the density *p* of cells

$$\begin{aligned} \frac{\partial p}{\partial t} = \frac{\partial }{\partial x}\left( -p\chi (S)\frac{\partial S}{\partial x} + D\frac{\partial p}{\partial x}\right) , \end{aligned}$$

where *S*(*x*, *t*) is the chemical concentration, and $\chi (S)$ describes the chemotactic sensitivity, and in general *S* may be prescribed to evolve according to some evolution equation. It is easily seen that if we assume that the chemical is independent of time, and choose the sensitivity to be of the form $\chi (S) = 1/S(X)$ or $\chi (S) = 2/S^2(X)$, the chemotactic equation become Eqs. (13) and (14) respectively.

To investigate how well the model is able to capture durotaxis, we chose biologically relevant parameters, and a sinusoidal stiffness function. This type of experiment has shown to produce clusters of cells on the “peaks” of high stiffness (Joaquin et al. 2016). Our models predicted cells to cluster, to different degrees depending on the parameter $\gamma$, which governs how much the adhesion lifetime is impacted by the stiffness. For example we showed that, to expect about 70–80% of the cells to be located in the stiffer regions, the lifetime of adhesion sites on the stiff side of the cell has to remain for 15–30% longer than the average lifetime of sites on the soft side of the cell. Although no experiments have yet investigated the lifetime of individual adhesions on a material with a stiffness gradient, we know from the study by Fusco et al. (2017) that the average lifetime of adhesions can be twice as long on stiff regions compared to soft regions. This suggests that a 15–30% difference in update frequency lies within a realistic range.

No visual difference between the two PDE models can be seen from Fig. 6c when $\gamma$ is chosen so that the models best fit the data. There is a small discrepancy at early and late times between model and data. The early time difference could possibly be due to effect such as cell–cell adhesion or a higher overall cell speed. The faster decay for large times could possibly be the result of crowding, which our models does not account for. In solutions of the PDE models shown in Fig. 6 we used a basic motility rate $\beta = 1$. If it is increased to 2, corresponding to an overall cell speed of about $40\,\upmu$m per hour, the fit becomes better. However, based on the result of the experimental study (Joaquin et al. 2016), a speed of $20\,\upmu$m per hour is more realistic.

To be able to disentangle how much of the observed drift of cells is the result of reinforcement of adhesion sites, one would need to measure the lifetime of individual adhesion sites within a cell, as well as the cell velocity. This would in turn be sufficient to estimate our model parameter $\gamma$, which describes how much of the displacement difference that influences adhesion site lifetimes. To our knowledge such a study has not yet been conducted. However, the lifetime of individual adhesion sites has been measured (Fusco et al. 2017), and it is well known that matrices with a range of stiffness profiles can be designed. It remains to study the velocity of cells and the lifetime of adhesion sites on a matrix with a stiffness gradient.

The 1D model of migrating cells might appear unrealistic for modelling 3D cell migration in a fibrous ECM. However, there are environments in which cell migration takes places in a highly organized ECM, along fibers. Examples include glioma cell migration in white matter tracts (Bellail et al. 2004), and stem cell migration in rats, in both transplantation into the auditory nerve and after spinal cord injuries (Palmgren et al. 2012; Tysseling et al. 2010). It is also believed that 1D cell migration along fibers is more similar to migration in 3D matrices than cell migration on typical 2D planar surfaces (Doyle et al. 2009, 2013). This makes 1D models suitable for modelling; they are more analytically tractable than 3D models, and they can provide results which more accurately translate into in vivo cell behaviour.

### Possible extensions

The purpose of our model is to investigate the impact of varying adhesion lifetimes on directed single cell migration. However, to more realistically model a large population of cells, one should incorporate interactions between cells, such as cell–cell adhesion or crowding effects, and interactions between cells and the ECM. For example, some cells have the ability to remodel the ECM, either through exerting physical forces, or through chemicals which break down the ECM. The former could be captured by explicitly modelling the ECM as an elastic material that deforms as cells exert forces on it. This could lead to interesting feedback between cells and the substrate. Another interesting property is the so-called strain-stiffening that can occur, which can alter the response of cells to the increased stiffness of the ECM close to large collections of cells e.g. close to tumours. Finally, should future experiments imply that reinforcements of adhesion sites play only a small role in the observed drift in durotaxis, our model can easily be extended to include for example asymmetric forces exerted by the cell or an asymmetric distribution governing new positions of adhesion sites, both of which would influence the drift velocity. One can also consider using a correlated random walk as the underlying mechanism of motility. In our model we assumed that the new position of an adhesion site was distributed normally around the current nucleus position, but one can make it more general by considering any other suitable distribution. However, in contrast to typical random walk models where only the first two moments of the jump kernel are of interest, using any other distribution in our model result in non-standard distributions of the nucleus jump distribution (data not shown). To the best of our knowledge, the normal distribution constitutes a special case where computations of all probability density functions are possible.

Understanding the interactions between cells and their environment is of fundamental importance and this paper will hopefully represent a step toward a deeper understanding of cell migration and its relation to substrate stiffness.

## Derivation of the advection–diffusion equation for the absolute relationship

We now assume that

$$\begin{aligned} \lambda _1 = \beta + \gamma \left( |u_1| - |u_2|\right) , \qquad \lambda _2 = \beta - \gamma \left( |u_1| - |u_2|\right) , \end{aligned}$$

which can be rewritten in terms of the stiffness, using $|u_i| = 1/E(x_i)$:

$$\begin{aligned} \lambda _1&= \beta + \gamma \left( \frac{1}{E(X-y)} - \frac{1}{E(X+y)}\right) , \\ \lambda _2&= \beta - \gamma \left( \frac{1}{E(X-y)} - \frac{1}{E(X+y)}\right) . \end{aligned}$$

We expand *E* in its Taylor series around *X* and obtain

$$\begin{aligned} \lambda _1&= \beta + \gamma \left( 2y\frac{E'(X)}{E(X)^2}+ \sum _{i =3,5,\ldots }^{\infty } T_i(X) y^i \right) \\ \lambda _2&= \beta - \gamma \left( 2y\frac{E'(X)}{E(X)^2} + \sum _{i =3,5,\ldots }^{\infty } T_i(X) y^i\right) \end{aligned}$$

where $T_i(X)$ is the *i*th coefficient in the Taylor series. As we showed in Sect. 3.2, under the rescaling of space and time, all terms of order $y^3$ and higher will go to zero, as $\varepsilon \rightarrow 0$, and is therefore not included in the proceeding calculation. We therefore have

$$\begin{aligned} \lambda _1&= \beta + \gamma \left( 2y\frac{E'(X)}{E(X)^2}\right) \\ \lambda _2&= \beta - \gamma \left( 2y\frac{E'(X)}{E(X)^2} \right) \end{aligned}$$

and $\varLambda = \lambda _1 + \lambda _2 = 2\beta$, along with mixture weights

$$\begin{aligned} w_1(X-y) = \frac{\lambda _1}{\varLambda }, \qquad w_2(X+y) = \frac{\lambda _2}{\varLambda }. \end{aligned}$$

We then obtain

$$\begin{aligned} f_{C}(r\, | \, X, y) = w_1(X-y) \frac{1}{\sqrt{2\pi \sigma ^2/4}}e^{-\frac{(r-y/2)^2}{2\sigma ^2/4}} + w_2(X+y)\frac{1}{\sqrt{2\pi \sigma ^2/4}}e^{-\frac{(r+y/2)^2}{2\sigma ^2/4}}. \end{aligned}$$

The unconditional distribution is then given by

$$\begin{aligned} f_{C}(r\, | \, X)&= \int _{0}^{\infty } \frac{1}{\sqrt{2\pi \sigma ^2/4}} \left( w_1(X-y)e^{-\frac{(r-y/2)^2}{2\sigma ^2/4}} \right) f_{Y}(y) \mathop {}\!\mathrm {d}y \\&\quad + \int _{0}^{\infty } \frac{1}{\sqrt{2\pi \sigma ^2/4}} \left( w_2(X+y)e^{-\frac{(r+y/2)^2}{2\sigma ^2/4}} \right) f_{Y}(y) \mathop {}\!\mathrm {d}y \\&= \frac{1}{2\beta }\int _{0}^{\infty } \frac{1}{\sqrt{2\pi \sigma ^2/4}} \left( \beta + \gamma y \frac{2E'(X)}{E(X)^2} \right) e^{-\frac{(r-y/2)^2}{2\sigma ^2/4}}\frac{\sqrt{6}}{\sigma \sqrt{\pi }}e^{-\frac{y^2}{2\sigma ^2/3}} \mathop {}\!\mathrm {d}y \\&\quad +\frac{1}{2\beta } \int _{0}^{\infty } \frac{1}{\sqrt{2\pi \sigma ^2/4}} \left( \beta - \gamma y \frac{2E'(X)}{E(X)^2} \right) e^{-\frac{(r+y/2)^2}{2\sigma ^2/4}} \frac{\sqrt{6}}{\sigma \sqrt{\pi }}e^{-\frac{y^2}{2\sigma ^2/3}} \mathop {}\!\mathrm {d}y \end{aligned}$$

Carrying out the integration we obtain

$$\begin{aligned} f_{C}(r\, | \, X) = \frac{1}{2\beta }\left( 2\beta + \gamma \frac{2E'(X)}{E(X)^2} r \right) \frac{1}{\sqrt{2\pi \sigma ^2/3}}e^{-\frac{r^2}{2\sigma ^2/3}}. \end{aligned}$$

Inside our transport equation we have the term $\varLambda$ as well, so we therefore cancel the factor $1/2\beta$. The transport equation is now

$$\begin{aligned} \frac{\partial q}{\partial t}(X,t)&= -\varLambda (X)q(X,t) \\&\quad \quad + \int _{\mathbb {R}}{q(X-r,t) e^{-\frac{r^2}{2\sigma ^2/3}}\frac{1}{\sqrt{2\pi \sigma ^2/3}}\left( 2\beta + \gamma r\frac{2E'(X-r)}{E^2(X-r)} \right) \mathop {}\!\mathrm {d}r}. \end{aligned}$$

We now perform the same rescaling of jump-size and time:

$$\begin{aligned} \sigma = \varepsilon \hat{\sigma }, \quad t = \frac{1}{\varepsilon ^2}\tau , \end{aligned}$$

for a small parameter $\varepsilon$. Writing $q(X,t) = q\left( X,\frac{1}{\varepsilon ^2}\tau \right) = \hat{q}\left( X,\tau \right)$ our equation becomes

$$\begin{aligned} \varepsilon ^2 \frac{\partial \hat{q}}{\partial \tau }(X,\tau )&= -\varLambda (X)\hat{q}(X,\tau ) \\&\quad \quad + \int _{\mathbb {R}}{\hat{q}(X-r,\tau ) e^{-\frac{(r/\varepsilon )^2}{2\hat{\sigma }^2/3}}\frac{1}{\varepsilon }\frac{1}{\sqrt{2\pi \hat{\sigma }^2/3}}\left( 2\beta + \gamma r\frac{2E'(X-r)}{E^2(X-r)} \right) \mathop {}\!\mathrm {d}r}. \end{aligned}$$

and we perform the change of variables $s\varepsilon = r$ and obtain

$$\begin{aligned} \varepsilon ^2 \frac{\partial \hat{q}}{\partial \tau }(X,\tau )&= -\varLambda (X)\hat{q}(X,\tau ) \\&\quad \quad + \int _{\mathbb {R}}{\hat{q}(X-s\varepsilon ,\tau ) e^{-\frac{s^2}{2\hat{\sigma }^2/3}}\frac{1}{\sqrt{2\pi \hat{\sigma }^2/3}}\left( 2\beta + \gamma s\varepsilon \frac{2E'(X-s\varepsilon )}{E^2(X-s\varepsilon )} \right) \mathop {}\!\mathrm {d}s}. \end{aligned}$$

We now expand $\hat{q}$ in its Taylor series around *X*, as well as $E'$ and *E* in series around *X* and obtain

$$\begin{aligned} \varepsilon ^2 \frac{\partial \hat{q}}{\partial \tau }(X,\tau )&= -\varLambda (X)\hat{q}(X,\tau ) \\&\quad \quad +\, \int _{\mathbb {R}}\left( \hat{q}(X,\tau ) -s\varepsilon \frac{\partial \hat{q}}{\partial X} + \frac{s^2\varepsilon ^2}{2}\frac{\partial ^2 \hat{q}}{\partial X^2}\right) e^{-\frac{s^2}{2\hat{\sigma }^2/3}}\frac{1}{\sqrt{2\pi \hat{\sigma }^2/3}} \\&\quad \quad \cdot \, \left( 2\beta + \gamma s\varepsilon \frac{E'(X)}{E^2(X)} - s^2\varepsilon ^2 \frac{d}{dx}\left( \frac{E'(X)}{E^2(X)} \right) + \mathcal {O}(\varepsilon ^3)\right) \mathop {}\!\mathrm {d}s. \end{aligned}$$

Dividing through by $\varepsilon ^2$, and taking the limit $\varepsilon \rightarrow 0$ we finally obtain the equation

$$\begin{aligned} \frac{\partial \hat{q}}{\partial \tau }(X,\tau )&= -\gamma \frac{\hat{\sigma }^2}{3} \frac{\partial }{\partial X}\left( q(X,\tau ) \frac{2E'(X)}{E^2(X)}\right) + \frac{\hat{\sigma }^2}{3}\beta \frac{\partial ^2 q}{\partial X^2}(X,\tau ) \end{aligned}$$

## Crank–Nicolson Scheme

For the PDE

$$\begin{aligned} \frac{\partial q}{\partial t}(x,t) = F\left( q,x,t,\frac{\partial q}{\partial x}, \frac{\partial ^2 q}{\partial x^2}\right) , \end{aligned}$$

the Crank–Nicolson method is the average of the forward Euler method at time *n* and the backward Euler method at time $n+1$

$$\begin{aligned} \frac{q_i^{n+1}-q_i^{n}}{\varDelta t} = \frac{1}{2}\left( F_i^{n+1}\left( x,t,q,\frac{\partial q}{\partial x},\frac{\partial ^2 q}{\partial x^2}\right) + F_i^{n}\left( x,t,q,\frac{\partial q}{\partial x},\frac{\partial ^2 q}{\partial x^2}\right) \right) , \end{aligned}$$

on a discretization of the spatial domain $x \in [-L,L]$ into *M* points of equal spacing *h*, $x_i = -L + hi$, and time domain discretized into *N* points of equal spacing $\varDelta t$, $t_n = n \varDelta t$.

## Distribution of adhesion site distances

In this section any lowercase *f* is a probability density function and any uppercase *F* a cumulative distribution function, with the corresponding random variable as a subscript.

Assume there are two adhesion sites, $x_1^0$ and $x_2^0$ at time $t = 0$. After *k* updates, the positions are $x_1^k$ and $x_2^k$. Denote the distance between the sites by $\varDelta x^k = x_1^k - x_2^k$, and the position of the nucleus by $\mu ^{k} = \left( x_1^k + x_2^k\right) /2$. When site *i* updates, its new position is given by

$$\begin{aligned} x_i^{k+1} = \mu ^{k} + W, \end{aligned}$$

where *W* is a normal random variable with mean 0 and variance $\sigma ^2$, and $i \in \{1,2\}$. The cumulative distribution function for $\varDelta x^k$ is

19 $$\begin{aligned} F_{\varDelta x^k}(z) = \mathbb {P}(\varDelta x^k \le z). \end{aligned}$$

We now consider the cumulative distribution after $k+1$ steps. Here we assume that the position of the adhesion sites at time-step *k* is $x_1$ and $x_2$, dropping the superscript *k*, and we denote by $x_1^+$ and $x_2^+$ the update position of site 1 and 2 respectively, depending on which one that updates. Assuming that site $i= 1$ updates with probability $\lambda _1$ and that site $i = 2$ updates with probability $\lambda _2$ , with $\lambda _1 + \lambda _2 = 1$ we obtain

$$\begin{aligned} F_{\varDelta x^{k+1}}(z)&= \mathbb {P}(\varDelta x^{k+1} \le z) \\&= \lambda _1 \mathbb {P}(x_1^+ - x_2 \le z) + \lambda _2 \mathbb {P}(x_1 - x_2^+ \le z), \end{aligned}$$

and re-writing $x_i^+ = \mu _k + W$, and using $\mu _k = (x_1 + x_2)/2$ we obtain

$$\begin{aligned} F_{\varDelta x^{k+1}}(z)&= \lambda _1\mathbb {P}\left( \mu _k + W - x_2 \le z \right) + \lambda _2\mathbb {P}\left( x_1 - \mu _k - W \le z \right) \\&=\lambda _1\mathbb {P}\left( \frac{x_1 + x_2}{2} + W - x_2 \le z \right) + \lambda _2\mathbb {P}\left( x_1 - \left( \frac{x_1 + x_2}{2}\right) - W \le z \right) \\&= \lambda _1\mathbb {P}\left( \frac{x_1 - x_2}{2} + W \le z \right) + \lambda _2\mathbb {P}\left( \frac{x_1 - x_2}{2} - W \le z \right) \\&= \lambda _1\mathbb {P}\left( \frac{\varDelta x^k}{2} + W \le z \right) + \lambda _2\mathbb {P}\left( \frac{\varDelta x^k}{2} - W \le z \right) . \end{aligned}$$

We now investigate the first term, and notice that

$$\begin{aligned} \mathbb {P}\left( \frac{\varDelta x^k}{2} + W \le z \right)&= \int _{-\infty }^{\infty } \int _{w = -\infty }^{z-x/2} f_{W,\varDelta x^k}\left( w,x\right) \mathop {}\!\mathrm {d}w \mathop {}\!\mathrm {d}x \\&= \int _{-\infty }^{\infty } \int _{w = -\infty }^{z-x/2} f_{W}\left( w \, | \, x\right) f_{\varDelta x^k}(x) \mathop {}\!\mathrm {d}w \mathop {}\!\mathrm {d}x \end{aligned}$$

Notice that the second equality is obtained from the fact that $\varDelta x^k$ and *W* are independent. If we differentiate with respect to *z* we obtain the pdf of distance between adhesion sites, and using the fact that *W* is independent of $\varDelta x^k$ we obtain

$$\begin{aligned} f_{\varDelta x^{k+1}}(z) = \int _{-\infty }^{\infty } f_{W}\left( z-\frac{x}{2}\right) f_{\varDelta x^k}(x) \mathop {}\!\mathrm {d}x \end{aligned}$$

and similarly for the second term we get

$$\begin{aligned} \mathbb {P}\left( \frac{\varDelta x^k}{2} - W \le z \right) = \int _{-\infty }^{\infty } f_{W}\left( -z+\frac{x}{2}\right) f_{\varDelta x^k}(x) \mathop {}\!\mathrm {d}x \end{aligned}$$

Together these two terms gives us the expression

$$\begin{aligned} f_{\varDelta x^{k+1}}(z) = \lambda _1 \int _{\mathbb {R}} f_{W}\left( z - \frac{x}{2} \right) f_{\varDelta x^k}(x) \mathop {}\!\mathrm {d}x + \lambda _2\int _{\mathbb {R}} f_{W}\left( -z + \frac{x}{2}\right) f_{\varDelta x^k}(x) \mathop {}\!\mathrm {d}x, \end{aligned}$$

but because $f_{W}$ is symmetric around 0, and $\lambda _1 + \lambda _2 = 1$ we end up with

$$\begin{aligned} f_{\varDelta x^{k+1}}(z) = \int _{\mathbb {R}} f_{W }\left( z - \frac{x}{2}\right) f_{\varDelta x^k}(x) \mathop {}\!\mathrm {d}x. \end{aligned}$$

It is easily shown that if $f_0$ is assumed to be a normal distribution with mean 0 and variance $\gamma ^2$, the distribution after the update of 1 adhesion site is normally distributed with mean 0 and variance $\sigma ^2 + \gamma ^2/4$. One can then ask the question, what is the variance of the distribution in equilibrium, i.e. when the variance does not change anymore. It is found simply by solving the equation

$$\begin{aligned} \sigma ^2 + \frac{\gamma ^2}{4} = \gamma ^2, \end{aligned}$$

namely $\gamma ^2 = 4\sigma ^2/3$. This means that the distribution of $\varDelta x^k$ converges to a normal distribution with variance $4\sigma ^2/3$ as $k \rightarrow \infty$, where $\sigma ^2$ is the variance for the distribution of new position of an adhesion site. The random variable *Y* defined as

$$\begin{aligned} Y^k = \left| \frac{\varDelta x^k}{2}\right| \end{aligned}$$

is a half-normal distribution obtained from the normal with zero mean and variance $\sigma ^2/3$.
